# Gastric volume estimation in children with cerebral palsy: which equation should be used?

**DOI:** 10.1016/j.bjane.2026.844841

**Published:** 2026-10-15

**Authors:** Mustafa Gökhan Bayram, Dinçer Fırat Şeker

**Affiliations:** a Balıkesir Atatürk City Hospital, Department of Anaesthesiology and Reanimation, Balıkesir, Türkiye; b Zonguldak Atatürk State Hospital, Anaesthesiology and Reanimation Clinic, Zonguldak, Türkiye

*Dear Editor*,

We read with interest the study reporting preoperative gastric ultrasound in 62 children with and without cerebral palsy.^1^ Children with cerebral palsy are under-represented in this literature, the scans were performed by a single trained operator, and the use of quantile regression is a welcome refinement. We write to ask for clarification of one point: the equation printed in the Methods does not appear to reproduce the volumes reported in the tables.

The Methods state that gastric volume was estimated as V=27.0+14.6×CSA(RLD)−1.28×age, the model validated by Perlas et al. in non-pregnant adults with a body mass index below 40 kg.m^-2^ and for volumes from 0 to 500 mL.^2^ Applied to the reported control medians (area 3.0 cm^2^, age 6.5 years, weight 31.0 kg) that equation returns 62.5 mL, or 2.0 mL.kg^-1^; applied to the cerebral palsy medians (4.0 cm^2^, 9.0 years, 29.0 kg) it returns 73.9 mL, or 2.5 mL.kg^-1^. The values reported in Table 1 of that article are 0.4 and 0.7 mL.kg^-1^. The equation also has a floor: with an intercept of 27.0 mL and an age term that cannot exceed 23.0 mL in children under 18 years, half of each group would necessarily be assigned at least 47.8 mL (controls) and 62.4 mL (cerebral palsy), against reported medians of 10.5 and 23 mL.

The model derived by Spencer et al. in 100 fasted children against endoscopically suctioned volumes fits the reported values more closely.^3^ That model is V=−7.8+3.5×CSA(RLD)+0.127×age in months. At the control medians it returns 12.6 mL, or 0.41 mL.kg^-1^; at the cerebral palsy medians, 19.9 mL, or 0.69 mL.kg^-1^. These calculations closely approximate the reported group medians of 0.4 and 0.7 mL.kg^-1^. The earlier study of children with cerebral palsy in this journal used the same equation, attributed to Spencer et al., together with the same two-diameter method of measuring antral area, and reported a median of 0.5 mL.kg^-1^.^4^ Three observations therefore point the same way: the numerical agreement at the group medians; the arithmetic floor of the printed equation, which is difficult to reconcile with the reported medians; and the identical two-diameter method and Spencer attribution in the earlier report in this journal. Taken together, the published results appear more consistent with the Spencer model, raising the possibility that the equation reported in the Methods may not correspond to that used in the analysis. Without the original dataset or analysis code this cannot be established, and we would be grateful if the authors could clarify which equation was applied.

The question matters directly for readers, while whether it has any implications for the analyses or conclusions can only be determined after clarification from the original authors. A clinician who applied the printed equation to a child would obtain a volume three to five times larger. At the reported lower quartile weight of 18 kg it exceeds 1.5 mL.kg^-1^ at any antral area above 1.6 cm^2^, below the reported lower quartile of 2.0 cm^2^, and applying the reported group median values returns 2.0 and 2.5 mL.kg^-1^, both above that threshold. We would not want to prejudge whether this is confined to the reporting of the Methods or bears on the analyses themselves; that is something only the authors can establish. We would add that the equation in the Methods carries a citation to reference 8 of that article, the systematic review by Van de Putte and Perlas;^5^ whichever equation was applied, that citation may be worth checking.

Two smaller points depend on the same clarification. First, the Discussion states that the formula used has not been validated in children. If the Spencer model was applied, that caveat is imprecise rather than wrong: the model has a paediatric derivation, although its own authors called for further validation, and it has not been studied in cerebral palsy. Second, the multivariable regression of estimated volume on age (Table 4 of that article) uses an outcome that is itself a function of age. If the Spencer equation was applied, age enters that outcome by construction at 0.127 mL per month, so roughly half of the reported +2.9 mL per year would follow from the estimator rather than from gastric physiology, the remainder reflecting the growth of antral area with age. Under the equation printed in the Methods the age term is negative and the contribution would run the other way. The coefficient is therefore difficult to interpret until the equation is confirmed, and the antral area, which is measured rather than derived, seems to us the more informative outcome, as it is for the primary analysis.

On comparability: Table 5 of that article shows that children with cerebral palsy taking continuous medication were scanned after a median fast of 2.0 hours, with clear liquid as the last intake in most, whereas controls were scanned after 8.0 hours with solid food. Both regimens comply with fasting guidance, and the authors note the difference in their Discussion; the point is one of comparability rather than adequacy, and the Abstract, which describes the groups as differing despite adequate fasting, does not carry that qualification. The comparison of unmedicated children against controls, whose fasting times were similar, is the more informative one.

None of this detracts from the study. The antral area is measured directly and requires no model, and the observation that the smallest areas recorded in controls were largely absent among children with cerebral palsy, supported by quantile regression, stands on its own. A clarification naming the equation actually applied would let readers apply these findings with confidence and allow a fair comparison with the earlier report in this journal.^4^

## Data availability statement

No new data were generated for this Letter. All calculations were based on data reported in the cited publications.

## Ethics approval

Not applicable. This Letter contains no original research on human subjects and analyses only data already published in the Brazilian Journal of Anesthesiology.

## AI assistance disclosure

The authors used Claude (Anthropic) to assist with drafting and language editing of this Letter. All calculations, scientific interpretation, references, and final content were verified by the authors, who take full responsibility for the manuscript.

## Authors’ contributions

MGB: Conception; Analysis of the published data; Drafting of the manuscript. DFŞ: Critical revision for important intellectual content. Both authors approved the final version.

## Funding

This research did not receive any specific grant from funding agencies in the public, commercial, or not-for-profit sectors.

## Conflicts of interest

The authors declare no conflicts of interest.
